# Stimuli-Responsive Hydrogels for Cancer Treatment: The Role of pH, Light, Ionic Strength and Magnetic Field

**DOI:** 10.3390/cancers13051164

**Published:** 2021-03-09

**Authors:** Fernanda Andrade, Maria Mercé Roca-Melendres, Esteban F. Durán-Lara, Diana Rafael, Simó Schwartz

**Affiliations:** 1 Drug Delivery and Targeting Group, Molecular Biology and Biochemistry Research Centre for Nanomedicine (CIBBIM-Nanomedicine), Vall d’Hebron Institut de Recerca, Universitat Autònoma de Barcelona, 08035 Barcelona, Spain; fernanda.silva@vhir.org (F.A.); mroca.3@alumni.unav.es (M.M.R.-M.); 2 Networking Research Centre for Bioengineering, Biomaterials, and Nanomedicine (CIBER-BBN), Instituto de Salud Carlos III, 28029 Madrid, Spain; 3 Department of Pharmacy & Pharmaceutical Technology, School of Pharmacy and Food Sciences, University of Barcelona, 08028 Barcelona, Spain; 4 Bio and NanoMaterials Lab, Drug Delivery and Controlled Release, Universidad de Talca, Talca 3460000, Maule, Chile; eduran@utalca.cl; 5 Departamento de Microbiología, Facultad de Ciencias de la Salud, Universidad de Talca, Talca 3460000, Maule, Chile

**Keywords:** hydrogels, pH-responsive, photosensitive, ionic strength-responsive, magnetic-responsive, drug delivery, cancer treatment, local treatment

## Abstract

**Simple Summary:**

Cancer remains as the world second leading cause of death. The severe side effects associated to high doses of chemotherapy and the development of drug resistance are major drawbacks for a successful cancer treatment. Therefore, new formulations that promote localized therapy at tumor sites are needed to improve the therapeutic outcomes and patient welfare. The use of hydrogels is a very promising alternative, since they can be composed by smart materials able to respond to external factors, changing their properties accordingly and promoting a localized drug delivery. As a result, a more specific, efficient, and less toxic local cancer treatment can be accomplished. In this context, the most important characteristics of hydrogels recent studies regarding the application of pH-, light-, ionic strength-, and magnetic-responsive hydrogels in cancer treatment are here presented.

**Abstract:**

Cancer remains as the second leading cause of death, worldwide. Despite the enormous important advances observed in the last decades, advanced stages of the disease remain incurable. The severe side effects associated to systemic high doses of chemotherapy and the development of drug resistance impairs a safe and efficiency anticancer therapy. Therefore, new formulations are continuously under research and development to improve anticancer drugs therapeutic index through localized delivery at tumor sites. Among a wide range of possibilities, hydrogels have recently gained special attention due to their potential to allow in situ sustained and controlled anticancer drug release. In particular, stimuli-responsive hydrogels which are able to change their physical state from liquid to gel accordingly to external factors such as temperature, pH, light, ionic strength, and magnetic field, among others. Some of these formulations presented promising results for the localized control and treatment of cancer. The present work aims to discuss the main properties and application of stimuli-responsive hydrogels in cancer treatment and summarize the most important advances observed in the last decades focusing on the use of pH-, light-, ionic strength-, and magnetic-responsive hydrogels.

## 1. Introduction

In 2018 the World Health Organization (WHO) reported an estimated 9.6 million cancer deaths, being the second leading cause of death worldwide. Lung, prostate, and colorectal are the most common types of cancer in men, while breast, colorectal and lung are the most common among women [1]. Unfortunately, despite all the advances achieved in the last decades in the pharmaceutical and biomedical fields, metastatic cancer is still considered an incurable disease. One of the major drawbacks regarding cancer treatment is related with the adverse effects associated with conventional chemotherapy [2,3,4]. Thus, sustained release local therapy emerged as promising alternative to reduce the systemic exposure and toxicity, thus increasing the therapeutic outcomes. In accordance to the National Cancer Institute definition, local therapy is conceived as a treatment directed to a specific organ or to a limited area of the body, embracing surgery, radiation therapy, cryotherapy, laser therapy, and topical therapy [5]. In recent years, a novel form of local cancer therapy using hydrogels have been proposed, owing to the favorable results achieved by these formulations in several other biomedical applications.

The present review is focused on stimuli-responsive hydrogels (pH-, photo-, ionic strength-, and magnetic-sensitive) proposed in the last 10 years as controlled release formulations for cancer treatment.

## 2. Hydrogels for Biomedical Applications

Different definitions for hydrogels have been suggested through the years. Often, they are described as a water-swollen and cross-linked polymeric network produced by the reaction/conjugation of one or more monomers. However, more recently hydrogels have been represented as three-dimensional networks which may absorb large amounts of water (from 10–20% up to thousands of times their dry weight) due to the presence of hydrophilic functional groups which fill the space among macromolecules and show high affinity for biological fluids [6]. 

The term hydrogel was firstly used by Wichterle and Lim in 1960 when they described a hydrogel based on poly-2-hydroxyethylmethacrylate (PHEMA), a synthetic biocompatible material useful for contact lens applications. In fact, in 1962 PHEMA lenses were firstly distributed in Western Europe, even though with limited success and acceptance. After a license buying agreement by the National Patent Development Corporation (NPDC) and a subsequent selling to Basuch and Lomb, that optimized the product, PHEMA lenses received Food and Drug Administration (FDA) approval [7]. Until today, hydrogels are routinely present in people using contact lenses, especially soft lenses based on silicon hydrogels. Moreover, hydrogels have been extensively used in biomedical applications regarding tissue engineering and wound healing [8,9]. Advanced dressings based on hydrogels proved to be more effective in wound healing since they maintain a moist environment at the application site that avoid the spread of fluids to other healthy areas of the skin. Nowadays, many of them are commercially available, such as DermaFilm^®^, Kaltostat^®^, Condress^®^, and Sofargen^®^ [10], being some of them filled with active ingredients such as iodine or zinc ions providing antimicrobial and cleansing properties, respectively [7]. Additionally, hydrogel-based formulations have been approved and used in the clinical practice for a variety of diseases. This includes Perseris^®^ a risperidone hydrogel for acute and chronic schizophrenia, Sublocad^®^ for buprenorphine induced analgesia, and Azasite^®^ with lidocaine hydrochloric acid for the treatment of bacterial conjunctivitis [11]. 

Owing to the demonstrated success of hydrogels in a variety of biomedical applications, it was speculated whether they could be used as drug delivery platforms for cancer treatment. Despite most of the research done in cancer treatment is dedicated to systemic and oral administration, local administration of drugs could be highly beneficial in cases of non-resectable or incomplete surgically removed tumors. In this context, the use of hydrogels, and especially stimuli-responsive hydrogels, has been proposed for in situ application at the tumor site/cavity, promoting a local sustained release of the drugs and a reduction of systemic exposure and off-target effects [12]. Moreover, administration of depot systems reduces the needs for repeated anticancer therapy cycles and the associated drawbacks to the patients and the economic impact in the health care systems [11]. 

Stimuli-responsive hydrogels are promising smart materials able to change conformation as a response to the surrounding environment variations like temperature, pH, light, ionic strength, and magnetic field [13]. This type of hydrogel has gained special importance due to the possibility to manipulate rheological behavior of the hydrogel according to the different tumor microenvironment conditions. In fact, cancer cells display several metabolic adaptations to ensure survival. Among them, glucose and nutrient uptake; production of lactic acid under aerobic conditions, and acclimatization to hypoxic and low-nutrient microenvironments. Moreover, an acidification of the extracellular milieu (low pH) and intracellular alkalization of the cytoplasm (high pH) occurs in cancer cells [14]. Thus, it is possible to take advantage of these characteristics to develop tumor targeted stimuli-responsive drug delivery systems. 

Over the years several hydrogel-based formulations have been designed for cancer diagnosis, prevention, and treatment, some of them enrolling clinical trials. Moreover, some have been granted with FDA and/or European Medicines Agency (EMA) approval, being translated into the clinical practice such as Lupron^®^ depot or Eligard^®^, two poly lactic-co-glycolic acid (PLGA)-based hydrogels for in situ delivery of leuprolide acetate against advanced prostate cancer [11]. Another example is TraceIT^®^, a polyethylene glycol (PEG) hydrogel microparticles containing covalently bound iodine, approved as tissue marker. After being injected it allows exact tumor visualization using magnetic resonance imaging (MRI), computed tomography (CT) and ultrasounds, thus providing more accurate identification of cancer cells before surgical removal. Moreover, it is also used to define more accurate dose planning in radiation therapy, avoiding unnecessary radiation of cancer-free areas. The product remains dimensionally stable for 3 months being fully absorbed after 6 months [15]. The feasibility and efficacy of TraceIT^®^ as organ spacer is under clinical evaluation in sparing vagina or prostate, in the treatment of rectal cancer patients, and also as a spacer between pancreas and duodenum in pancreatic cancer patients [16]. Another example is SpaceOAR^®^, an FDA approved PEG-based hydrogel organ spacer used to prevent rectum injury during radiation therapy sessions in prostate cancer patients [17]. 

## 3. Classification of Hydrogels

The majority of the developed hydrogels for biomedical applications are based on polymers with medium to high molecular weight; however, in recent years, low molecular weight self-assembling systems have been described and proposed. These systems are generally based on low molecular weight gelators, with especial focus on peptides that self-assemble into long, anisotropic structures, most commonly fibers. At a certain concentration, these fibers crosslink forming the gel network. Depending on the gelators properties they could be used to develop stimuli-responsive hydrogels [18,19]. Low molecular weight self-assembling peptide-based materials were the focus of a recent paper reviewing its properties and potential biomedical application [18]. In the present review we will focus on stimuli-responsive polymer-based hydrogels intended for cancer treatment. In Table 1 are presented some of examples of polymers used in the development of hydrogels.

Hydrogels can be classified according to different parameters including: (i) composition (homo or copolymeric), (ii) network size (macrogels, microgels, nanogels), (iii) electrical charge (non-ionic, cationic, anionic, amphoteric or zwitterionic), (iv) crosslinking (physical or chemical), among others [20].

**Figure 1:**
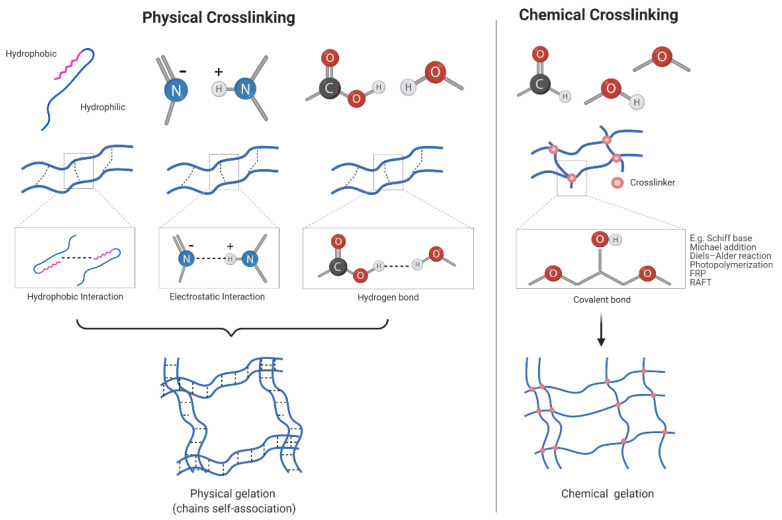
Schematic representation of hydrogels formation via chemical and physical crosslinking. RAFT—reversible addition-fragmentation chain transfer polymerization, FRP—free radical polymerization. O—oxygen, N—Nitrogen, C—Carbon, H—Hydrogen. Created with BioRender.com (accessed on 15 January 2021).

Regarding the type of crosslinking, hydrogels can be prepared via physical or chemical crosslinking (Figure 1). When a physical crosslinking is used, there is no formation of covalent bonds and the hydrogels present phase-reversibility. The main physical interactions between polymer chains described in physically crosslinked hydrogels are based on hydrogen bonding, ionic/electrostatic interactions, self-assembly of amphiphilic polymers by hydrophobic interactions, crystallization, protein interactions, and metal coordination [21]. For example, polyacrylic acid and polymethacrylic acid forms complexes with PEG through a hydrogen bonding. They are only formed when exists a protonation of carboxylic acid groups, which would result in the formation of a pH-sensitive hydrogel [22]. Another example is alginate, a natural polymer that jellifies through ionic interactions with divalent cations such as calcium (Ca^2+^) or magnesium (Mg^2+^) [21]. On the other hand, amphiphilic polymers such as poloxamers suffer thermally induced phase transition forming hydrogels due to hydrophobic interactions above lower critical solution temperature (LCST) [23]. Polyvinyl alcohol (PVA)-based hydrogels are an example of crystallization induced physical crosslinking as polymer crystallization and gelation occurs when the solution repeatedly undergoes freeze-thawing cycles [24].

The biggest drawback of physical crosslinking hydrogels relies on their possible lack or reduced stability under physiological conditions. Therefore, chemical crosslinking is being preferred for in vivo applications [22]. 

Chemically crosslinked hydrogels are more stable under physiological conditions and exhibit excellent mechanical properties owing to the covalent bonds in-between the polymer chains. Additionally, their degradation behavior can be tunable, even though they are generally irreversible. A potential disadvantage compared to physically crosslinked hydrogels is the frequently required use of organic solvents and catalysts that often raise biocompatibility and environmental concerns [21].

Covalent bonds in chemically crosslinked hydrogels are generally promoted by click chemistry including Schiff base formation, Michael addition or Diels–Alder reaction, photopolymerization, free radical polymerization (FRP), reversible addition-fragmentation chain transfer (RAFT) polymerization or enzyme catalyzed reactions [21].

Due to the presence of certain chemical groups such as NH_2_-, COOH-, OH- in the skeleton of hydrophilic polymers, the amine-carboxylic acid, isocyanate-OH/NH_2_ reaction or Schiff base formation are commonly used to create a covalent linkage between polymers to form the hydrogel network. Schiff base is commonly used for hydrogels formation and generally obtained via the nucleophilic attack of amines on the electrophilic carbon atoms of aldehydes or ketones. For example, glycol chitosan have been crosslinked with PEG or poly(N-isopropylacrylamide)-co-poly(acrylic acid) forming promising hydrogels for anticancer drug delivery [21]. In photopolymerization crosslinking, acrylate derivatives (e.g., poly (ethylene glycol) diacrylate (PEGDA) and glycidylmethacrylate (GMA)) are generally used as pre-polymers due to the presence of unsaturated groups. These groups react with photo-initiators (UV or visible light) like Irgacure 2959, lithium phenyl-2,4,6-trimethylbenzoylphosphinate (LAP) or Eosin-Y, among others. In the presence of a light source, photo-initiators are cleaved by photons forming free radical molecules that react with the vinyl bonds in the pre-polymer promoting the crosslink between the polymer chains [25]. Enzyme catalyzed crosslinking can be used, for example, to create of PEG-based hydrogels using a transglutaminase, which catalyzes the reaction between the γ-carboxamide group of the PEG-functionalized with glutaminyl groups and the ε-amine group of poly(lysine-co-phenylalanine) resulting in the formation of an amide bond [26].

To avoid the irreversibility while maintaining the higher mechanical strength of chemically crosslinked hydrogels, dynamic covalent chemistry is being applied to generate hydrogels via both physical and chemical crosslinking [21]. For example, boronate esters prepared from boronic acids and 1,2- or 1,3-diols in aqueous solutions results in reversible covalent bonds. Importantly, the strength and reversibility of the bond largely depends on the pH of the solution and the pKa of the boronic acid used as the boronate ester formation is favored at pH values above the pKa of the boronic acid. By changing the boronic acid derivative and the 1,2- or 1,3-diols used, is possible to produce reversible pH-sensitive hydrogels with tunable mechanical properties [21].

## 4. Stimuli-Responsive Hydrogels

Some hydrogels can be sensitive to environmental stimulus such as temperature, pH, light, ionic force or pressure, suffering sol-to-gel transitions that can allow controlled gelation and drug release at specific sites. On the other hand, non-sensitive hydrogels swell due to water absorption without responding to environmental changes. Thermo-responsive hydrogels are the most commonly used being the main subject of a bibliographic revision performed by our group (under publication). Here we report some of the pH-, light-, ionic strength-, magnetic-sensitive, as well as examples of dual responsive hydrogels developed in recent years for cancer treatment.

### 4.1. pH-Sensitive Hydrogels

Cancer cells are characterized by the acidification of the extracellular milieu and increased alkalization of the cytoplasm. Some studies suggest that alkaline intracellular pH increases glycolysis, promotes cellular adaptation to hypoxia and maximizes cancer cell proliferation [14]. Taking advantage of the pH alteration at the tumor environment, new pH-sensitive hydrogels have been developed in order to release the cytotoxic drug exclusively in the cancer cells area, reducing the occurrence of adverse side-effects in healthy tissues. 

**Figure 2:**
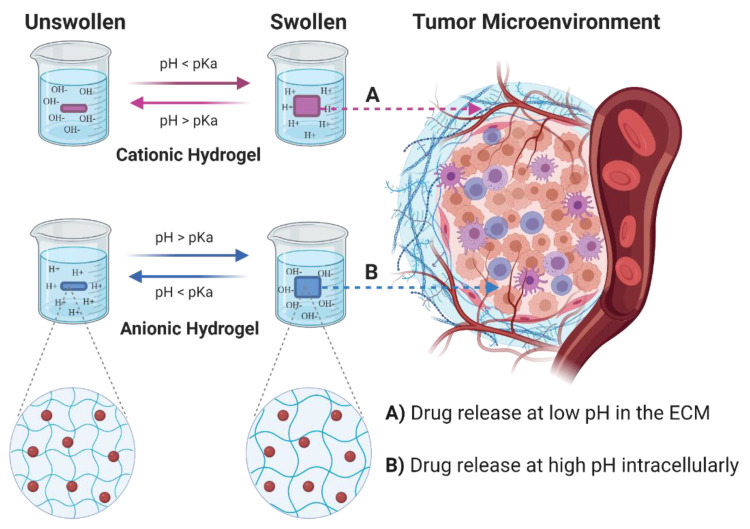
Schematic representation of pH-sensitive hydrogels swelling. Swelling of the hydrogel occurs via ionization of the pendant groups with consequent increase hydrophilic nature of the polymer chains and electrostatic chain repulsion. ECM—extracellular matrix, H^+^—Hydrogen ions, OH^−^—Hydroxide ions. The red circles represent the drugs loaded in the hydrogels. Created with BioRender.com (accessed on 15 January 2021).

To create pH-sensitive hydrogels, ionizable groups including amines, carboxylic acids, imines, etc., can be introduced in their structure. These groups, depending on the pKa values and the environmental pH, present the capability to donate or accept protons and undergo alterations of their physical and chemical properties [27,28]. Cationic hydrogels swell at low pH (pH < pKa) due to protonation of amino/imine groups and consequent repulsion of the positively charged moieties on the polymer chains. On the contrary, anionic hydrogels swell at higher pH (pH > pKa) due to ionization of the acidic groups and chain repulsion (Figure 2) [29]. pH-sensitive conformational changes of polymer can occur via: (a) destabilization (via collapse or swelling), (b) dissociation, and (c) alteration of the drug/vehicle partition coefficient. Conformational changes or solubility alterations will affect the release of encapsulated drugs. The kinetics of release could be controlled by manipulating the degree and ratio of the conformational changes [27,28]. For example, cationic hydrogels release the drug when swell at low pH (Figure 2A), while anionic hydrogels release the drug at higher pH values (Figure 2B). For this reason, anionic hydrogels are preferred for controlled intracellular drug delivery in tumor cells, while cationic hydrogels are preferred for delivery at the extracellular matrix in tumor tissues [29,30]. 

pH-sensitive hydrogels can be also produced by formation of polyelectrolyte complexes between anionic (e.g., alginate, dextran) and cationic (e.g., chitosan) polymers, thus avoiding the use of chemical and possibly toxic crosslinkers [29,31,32]. The use of acid-labile chemical bonds that are stable at physiological (neutral) pH and degrade or hydrolyze at low pH values (e.g., tumor microenvironment) is also a good strategy to produce this “smart” hydrogels, especially in the field of cancer treatment [27]. 

One of the most used polymers to produce pH-responsive hydrogels is chitosan. It is a natural, non-toxic, biodegradable, and biocompatible polymer obtained from chitin. However, the limited solubility in water and other organic solvents is its weakness. To overcome this, different derivatives of chitosan presenting a good solubility in physiological conditions such as N-trimethyl chitosan, N-carboxymethyl chitosan, or N-carboxyethyl chitosan (CEC) have been developed [29,33]. For example, amine groups from CEC and benzaldehyde groups from PEGDA create a dynamic covalent Schiff-base linkage forming a doxorubicin-loaded hydrogel with self-healing performance [33]. Moreover, the hydrogel presented adequate physical, rheological, and biological properties for the targeted delivery of doxorubicin in hepatic cancer. Of note, since the pKa value of D-glucosamine residue of chitosan is about 6.2~7.0, the hydrogel promoted drug release at low pH values (92% of doxorubicin was released at pH 5.5 over 42% at pH 7.4 after 7 days), due to the decomposition of the Schiff-base (14% mass loss at pH 7.4 and 40% at pH 5.5) and swelling/increase in pore size. More importantly, at low doses, the hydrogel loaded with doxorubicin significantly inhibited the proliferation of HepG2 cells when compared to the free drug [33]. A similar behavior was obtained with a CS/PVA hydrogel loaded with 5-fluoracil. The hydrogel retained the drug at pH 7.4, thus preventing possible drug release to normal cells and the consequent side effects, promoting a continuous and controllable drug release at pH 5 [34].

Many other natural polymers (e.g., alginate, cellulose, guar gum, carrageenan, dextran, xanthan, among others) and synthetic polymers (e.g., polyamines like poly(acrylamide), poly(N,N′-dimethyl aminoethyl methacrylate) or poly(ethylene imine), acrylic derivatives like poly(acrylic acid), sulfonic acid derivatives like 2-acrylamido-2-methylpropylsulfonic acid, sulfonamides like sulfamethazine, pyridine derivatives like poly(2-vinyl pyridine), imidazole derivatives like poly(N-vinyl imidazole), PEG, PVP, PLA, among others) have been proposed for production of pH-sensitive hydrogels [29,35].

Beyond injectable hydrogels, pH-responsive hydrogels are also an interesting tool for oral delivery of anticancer drugs as the gastrointestinal tract is characterized by variations in pH. Therefore, it is possible to tune formulations to promote the release of drugs in affected regions of the digestive tract [35]. As example, different pH-responsive nanogels based on poly(methacrylic acid-g-polyethylene glycol-co-hydrophobic monomer) were developed and assessed as platforms for intestinal delivery of doxorubicin for colorectal cancer treatment [36]. The different hydrophobic monomers used to adjust pH-responsiveness were tert-butyl methacrylate (tBMA), n-butyl methacrylate (nBMA), n-butyl acrylate (nBA), and methyl methacrylate (MMA), while polyethylene glycol was added to provide better integrity of the hydrogel and confer P-gp efflux inhibition. Among the formulations the one composed by poly(methacrylic acid-g-polyethylene glycol-co-methyl methacrylate) presented the best performance, namely, doxorubicin loading and release at simulated intestinal pH, and transport across intestinal epithelia (Caco-2 cell monolayers) [36].

pH-sensitive hydrogels can be also applied for gastrointestinal protection and regulate acute radiation syndrome, a consequence of tumor radiation therapy. A hydrogel constituted by polycaprolactone grafted with poly(methacrylic acid-co-ethyl acrylate) (PCL-g-MAC) was proposed for intestinal delivery of amifostine, and FDA approved radioprotective agent [37]. MAC is an FDA approved excipient for enteric coating due to its pH-responsiveness, while PCL was used to afford hydrophobicity to the system, improving hydrogel stability and performance. The hydrogel protected amifostine from gastric degradation (pH 1.2 release of 36.5% for 5.5 h), releasing the drug only at pH 7.4 (70.7% release at 0.6 h), as the pKa of the carboxyl groups is around 4.5, the hydrogel tends to dissociate and swell for pH > 4.5. Moreover, in vivo studies suggested that the formulation promotes protection and enhances survival upon γ-radiation by inhibiting hematopoietic cell apoptosis and accelerating cell proliferation, and also by reducing weight loss [37]. Some examples of pH-sensitive hydrogels developed in the last decade proposed as drug delivery systems for cancer treatment are summarized in Table 2.

### 4.2. Photosensitive Hydrogels

**Figure 3:**
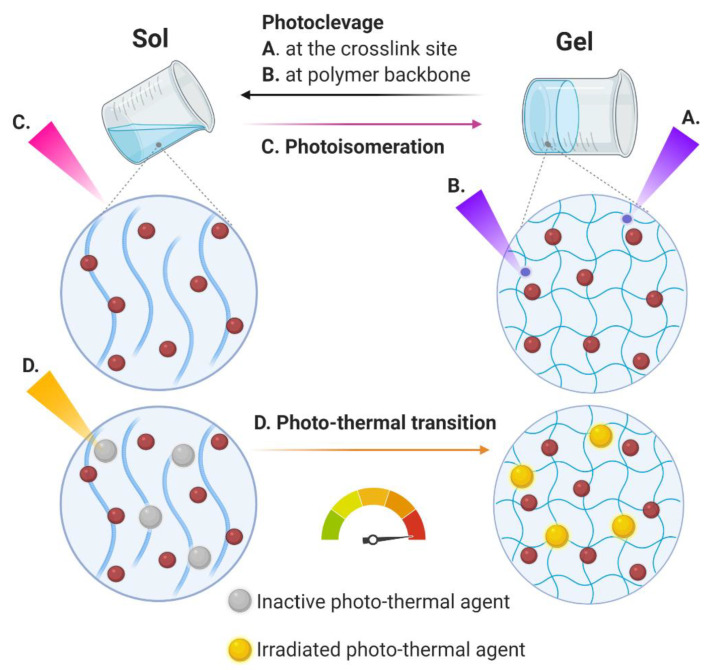
Schematic representation of photosensitive hydrogel. After exposure to light, the formulation suffers a chemical and/or physical alteration including (**A**,**B**) photoclevage, (**C**) photoisomeration, and (**D**) photo-thermal transition. In the case of photo-thermal transition, the increase in the temperature promoted by the activation of photo-thermal agents could lead to a sol-to-gel or a gel-to-sol transition, accordingly the nature of the thermo-responsive polymer. The red circles represent the drugs loaded in the hydrogels. Created with BioRender.com (accessed on 15 January 2021).

Photosensitive hydrogels suffer a chemical and/or physical alteration as a consequence to light exposure (UV, visible or near infrared (NIR)) (Figure 3). An incident light triggers modifications such as free-radical polymerization reaction, chemical linkage cleavage or isomerization, volume changes via swelling or shrinkage, etc. [51,52]. By application of light at the desired site of action is possible to promote a spatial and temporal controlled drug release avoiding systemic exposure. There are mainly three strategies used in the development of light-responsive hydrogels as drug delivery systems: (i) photochemical cleavage of or crosslinking points (Figure 3A) polymer backbones (Figure 3B), (ii) photo-induced isomerization of specific motifs that alter gel properties like the crosslinking density, charging state, or hydrophilicity (Figure 3C), and (iii) incorporation of photothermal agents that generate heat through irradiation and induce phase transition of thermo-responsive hydrogels (Figure 3D) [53]. Photoisomerization usually originates reversible hydrogels, while photocleavage is usually irreversible. Ruthenium, coumarin nitrophenyl, or o-nitrobenzyl derivatives, are examples of photocleavable compounds used in the development of hydrogels, while azobenzene and spiropyran promote isomerization under light exposure [51,54]. On the other hand, photo-thermal agents include porphyrins, cyanines, gold, silver, oxide, and carbon nanoparticles [51]. Regarding photothermal induced hydrogels, NIR-responsive hydrogels are of great interest due to their deep tissue penetration and harmlessness; however, overheating and consequent tissue damage is one of the main concerns that must be properly addressed regarding its biomedical application [51].

As an example of photo-thermal hydrogels, black phosphorus nanosheets incorporated into an agarose hydrogel loaded with doxorubicin demonstrated to be a promising platform for cancer treatment [55]. NIR irradiation promote heat production by phosphorus nanosheets and gel-to-sol transition of the agarose hydrogel by hydrolysis that triggers temporal and spatial doxorubicin release. The hydrogel showed to be biocompatible in vitro in MDA-MB-231 breast cancer cells, A549 lung carcinoma cells, HeLa cervical cancer cells, and B16 melanoma cells. In vivo experiments demonstrated that this system is capable of accurately controlling the release of drugs and eradicate the tumor in contrast to animals treated with free dox or the non-irradiated hydrogel. Agarose is approved by FDA and black phosphorus degrades into nontoxic phosphate and phosphonate that could be easily excreted through urine, which favors the clinical translation of the system [55]. Sodium selenite (Se)-directed crosslinked hydrogels based on hyaluronic acid-dopamine, loaded with indocyanine green (ICG) as photo-thermal agent, were developed for local therapy of breast cancer [56]. After NIR irradiation, ICG promote an increase in the temperature superior to 7 °C, responsible for both in vitro and in vivo photo-thermal activity against MDA-MB-231 breast cancer cells and in tumor-bearing BALB/c nude mice models, respectively. Additionally, the system enjoyed from the combinational effect of ICG and pro-oxidant effect of Se, resulting in a significant tumor inhibition without significant toxic effects [56].

The use of photosensitizer compounds incorporated into hydrogels for anticancer photodynamic therapy is also being proposed. For example, phthalocyanine zinc (ZnPc) was used both as photo-initiator and photosensitizer in the formulation of a hybrid hydrogel containing PEGDA, PEG 400 and phosphotungstic acid [57]. When irradiated with NIR laser light, ZnPC become excited forming a singlet oxygen (^1^O_2_), that promotes a strong decrease on HeLa cells. Moreover, the hydrogel presents a good biocompatibility and offers the possibility to be loaded with anticancer drugs for a combined and synergic therapy [57]. More recently, similar results have been obtained with a PEGDA-based hydrogel containing methylene blue sensitized mesoporous titanium (IV) oxide (TiO_2_) nanocrystals prepared in situ via NIR photopolymerization [58]. TiO_2_ was used as photo-initiator and photosensitizer while methylene blue works as photosensitive additive to improve the effects of TiO_2_ [58].

Combinatory effects of photo-thermal activity and photodynamic therapy has also been investigated. For example, a biocompatible agarose-based hydrogel that incorporates sodium humate (SH) as photothermal agent, chlorin e6 (Ce6) as photosensitizer, and manganese oxide (MnO_2_) for the catalytic decomposition of H_2_O_2_ and modulation of hypoxia at the tumor microenvironment, associated to treatment resistance [59]. Under NIR irradiation, SH promote an increase in the temperature that besides the photothermal activity, promote degradation of the agarose gel forming H_2_O_2_. This will be decomposed into O_2_ which will convert into ^1^O_2_ by the released Ce6. In vivo studies in 4T1 BALB/c mice bearing demonstrated a strong anticancer activity of the system with a significant tumor regression (TGI of 93.8%), without causing toxicity to the animals. Accordingly, this hydrogel should be considered as a promising platform for tumor microenvironment modulation and cancer therapy [59].

### 4.3. Ionic Strength and Magnetic-Responsive Hydrogels

Besides temperature, pH and light, which are the most commonly used stimuli for development of sensitive hydrogels, there are other parameters such as ionic strength or magnetic field that can also modulate the physical and chemical attributes of stimuli-responsive hydrogels.

**Figure 4:**
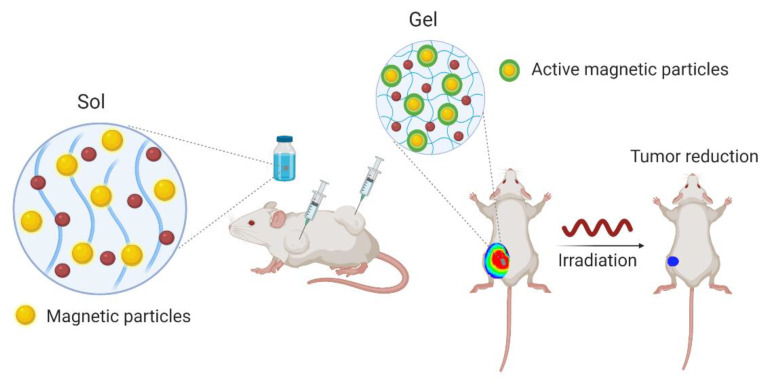
Schematic representation of magnetic-responsive hydrogels. They incorporate nanoparticles, which under a magnetic field vibrate due to their paramagnetic properties. A temperature increase is induced triggering drug release. The red circles represent the drugs loaded in the hydrogels. Created with BioRender.com (accessed on 15 January 2021).

Magnetic-sensitive hydrogels are generally composed by hydrogels incorporated with iron oxide nanoparticles possessing paramagnetic properties (Figure 4). These vibrate under exposure to a magnetic field and can dramatically increase local temperature promoting a therapeutic efficacy by thermal-ablation mechanisms. Moreover, these systems are generally associated with thermo-sensitive hydrogels in which temperature increase triggers drug release, thus promoting a synergic efficacy of thermal and chemotherapeutic cytotoxicity. As NIR, this strategy enjoys of spatial and temporal activity with low invasiveness and deep tissue penetration [35].

As an example, ferromagnetic vortex-domain iron oxide nanorings (FVIOs) were incorporated into chitosan-PEG-based hydrogel loaded with doxorubicin. Both in vitro and in vivo studies demonstrated a synergic therapeutic efficacy of chemotherapeutic mechanism of doxorubicin and thermal activity of FVIOs upon application of a magnetic field. After tumor surgical removal and formulation administration was possible to observe almost complete inhibition of tumor growth 21 days post-surgery, showing the efficacy of the system in reducing tumor recurrence [60]. Further, another example is a magnetic-sensitive hydrogel composed by PEGylated Fe_3_O_4_ nanoparticles and α-cyclodextrin stabilized by a PEGylated phospholipid encapsulated with paclitaxel and doxorubicin [61]. The rheological properties of the hydrogel allow to easily inject the hydrogel at the surgical site after tumoral resection, which facilitates local delivery of its dual cargo due to magnetocaloric gel-to-sol transition under application of a magnetic field. In vivo experiments in 4T1-tumor bearing Balb/c mice demonstrated an improvement in the survival rate and reduction in tumor recurrence of animals treated with the proposed hydrogel [61].

**Figure 5:**
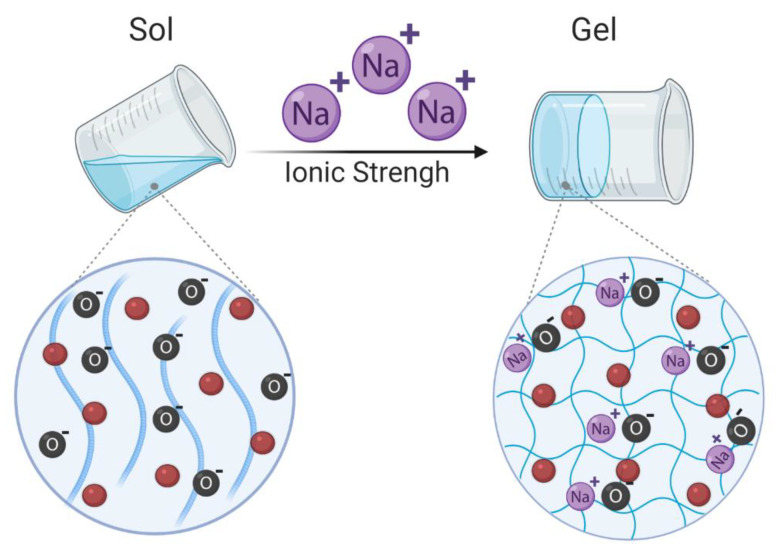
Schematic representation of ionic strength responsive hydrogels. Swelling of the hydrogel occurs when the ionic strength of surrounding medium increases. O—Oxygen, Na^+^—Sodium. The red circles represent the drugs loaded in the hydrogels. Created with BioRender.com (accessed on 15 January 2021).

Ionic strength-sensitive hydrogels refer to hydrogels that suffer conformational changes in response to cations such as K^+^, Na^+^, and Ca^2+^ (Figure 5). They are generally produced with ionizable and zwitterionic polymers such as alginate, deacetylated gellan gum, carboxymethyl dextran, poly(acrylic acid), poly(itaconic acid), sulfobetaine and carboxybetaine derivatives, polypeptides, among others [11,62]. Up to now, few ionic strength-responsive hydrogels as platforms for cancer treatment have been proposed. As an example, poly (L-glutamic acid-co-L-lysine)-based hydrogels loaded with doxorubicin have shown an ionic strength sensitivity by an increase in the swelling ratio of the hydrogel proportional to the increase of the ionic strength of the surrounding medium [63]. At high ionic strength, Cl^−^ and Na^+^ shield the NH^3+^ and the COO^−^ polypeptidic groups preventing electrostatic interactions and promoting hydrogel swelling. The presented formulation also presented pH and enzymatic sensitiveness [63].

### 4.4. Dual-Responsive Hydrogels

Dual-responsive hydrogels, namely thermo- and pH sensitive hydrogels have attracted high attention in the biomedical field due to their wide range of possible applications and functionalities. In the case of cancer, it is of great interest a system able to be easily injected that suffer a sol-to-gel transition in situ in response to body temperature and promote drug release at specific tumoral pH. For instance, a dual thermo- and pH-sensitive injectable hydrogel based on chitosan with poly(N-isopropylacrylamide-co-itaconic acid) was developed for delivery of doxorubicin for breast cancer treatment [64]. Due to the protonation of the amino groups of chitosan at acidic pH, doxorubicin was released more rapidly at the pH 5.5 than at pH 7.4. The in vitro cytotoxicity studies demonstrated cytocompatibility of the aforementioned hydrogels and the enhancement of the doxorubicin activity against MCF-7 breast cancer cells [64]. Another formulation with similar properties was proposed based on a nano-hydrogel of lysine-modified poly(vinylcaprolactam) loading doxorubicin [65]. Lysine was conjugated with poly(vinylcaprolactam) via reversible addition-fragmentation chain transfer (RAFT) polymerization while doxorubicin was conjugated through Schiff-base reaction, responsible for the pH sensitiveness property. The maximum release of doxorubicin for 72 h was observed at the simulated tumor microenvironment, namely 40 °C and pH of 5 [65]. A crosslinker-free thermo- and pH-responsive hydrogel based on poly(ethylene glycol) methyl ether methacrylate and acrylic acid was proposed for oral delivery of 5-fluoracil in colorectal cancer treatment [66]. The swelling behavior of the proposed hydrogels shown to be highly dependent on the pH and temperature, with higher swelling at low temperatures, thus retaining the drug at 37 °C, being the release profile mainly dependent on the pH. Contrary to what happens at pH 1.2 and 4, the hydrogel swells due to the electrostatic repulsion between the ionized carboxyl groups at pH > 6 and release the drug (<20% at pH 1.2 and ~50% at pH 7.4 for 37 °C). This avoids drug release at the gastric environment and promotes drug availability in the intestinal lumen. Moreover, the developed hydrogel shown to be biocompatible and promote a controlled release of 5-fluouracil presenting pharmacological activity against HepG2 tumor cells [66]. 

More recently, it was proposed a system for co-delivery of granzyme B (GrB) and docetaxel (DOC) loaded in pH-sensitive mini micelles incorporated in a thermo-sensitive hydrogel [67]. Micelles were composed by poly (γ-glutamic acid)-poly(L-histidine) (PGA-PLH) and the hydrogel composed by poly (ethylene glycol)-poly(γ-ethyl-L-glutamate) diblock copolymer (mPEG-b-PELG). After release from the hydrogel by proteinase degradation, micelles shown to be able to penetrate in the tumor and release the cargo at pH 5.5. Moreover, it was demonstrated that both drugs were able to maintain activity after lysosomal escape and promote a synergic antitumor efficacy as improve the tumor inhibition capacity in B16 tumor-bearing female C57BL/6 mice model [67]. 

Even most of the developed dual-responsive hydrogels are pH and thermo-sensitive, other types of dual responsiveness have been proposed. As an example, a novel formulation of a photo and thermo-responsive multicompartment hydrogel for cancer treatment [68]. The formulation is based on the triblock copolymer poly(N-isopropylacrylamide)-b-poly(4-acryloylmorpholine)-b-poly(2-((((2-nitrobenzyl)oxy)carbonyl)amino)ethyl methacrylate) (PNBOC-b-PNAM-b-PNIPAM), which forms a PNBOC-cored micelles and consequently hydrogels after being exposed to high concentrations and temperatures. Because of both hydrophilic and hydrophobic domains, it was loaded with gemcitabine and doxorubicin. After administration, the hydrogel suffers a sol-to-gel transition retaining both drugs at the administration site. A significant drug release is obtained when UV irradiation is applied at the desired site of action due to sol-to-gel transition as a consequence of crosslinking of micellar cores and hydrophobic-to-hydrophilic transition [68].

## 5. Conclusions

The design of new strategies to obtain new and effective local cancer treatments, to improve the therapeutic outcome and to reduce the systemic toxicity associated with the conventional drugs, are of utmost importance. Hydrogels bring the possibility to create an in situ sustained and controlled delivery system of anticancer drugs at the tumoral site. In particular, stimuli-responsive hydrogels open a wide range of new options to specifically target the tumor site. Drug release at tumor site can be modulated by taking advantage of the specific physiologic characteristics in the tumor microenvironment, namely the differential extracellular and intracellular pH values. Moreover, the application of light, magnetic field, or even alterations in the ionic strength, can regulate the rate and location of drug release. Despite not being widely explored, these are promising approaches to the future of local cancer therapy, especially in the case of non-surgically resectable tumors. It is expected that in a near future new smart materials and new formulations designed and validated with this propose will hopefully enroll in clinical trials and reach the clinical practice.

## Figures and Tables

**Table 1:** Chemical structure of some of the most commonly used polymers in hydrogels production. PEG—polyethylene glycol, PEGDA—poly (ethylene glycol) diacrylate, PLGA—poly lactic-co-glycolic acid, PVA—poly(vinyl alcohol). The oxygen (O) atoms are highlighted in red and the nitrogen (N) atoms in blue.

Polymer	Chemical Structure
Acrylamide Polymer base for a variety of derivatives including polyacrylamide, bisacrylamide, N-Isopropylacrylamide, N-N′-dimethylacrylamide	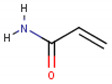
Methacrylate Polymer base for a variety of derivatives including poly-2-hydroxyethylmethacrylate, poly(N,N′-dimethyl aminoethyl methacrylate), tert-butyl methacrylate, n-butyl methacrylate, n-butyl acrylate, and methyl methacrylate	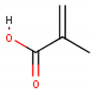
PLGA (x = number of units of lactic acid and y = number of units of glycolic acid)	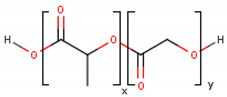
PEG (*n* = number of units of ethylene glycol)	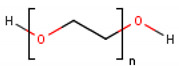
PEGDA (*n* = number of units of ethylene glycol)	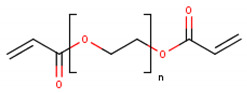
Chitosan (*n* = number of units of β-(1→4)-linked D-glucosamine and N-acetyl-D-glucosamine)	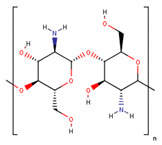
Alginate (x = number of units of (1,4)-linked β-D-mannuronate and y = number of units of α-L-guluronate)	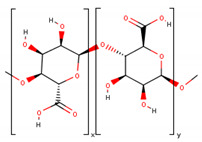
PVA (*n* = number of units of vinyl alcohol)	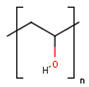
Poloxamer (x = number of units of ethylene glycol and y = number of units of propylene glycol)	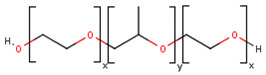

**Table 2:** Examples of pH-sensitive hydrogels for cancer therapy. n.a.—not applicable; β-CD—β-cyclodextrin; 5-FU—5-fluorouracil; DOX—doxorubicin; FACS—fluorescence-activated cell sorting; PTX—paclitaxel.

Date	Drug	In vitro model	In vivo model	Hydrogel formulation	Highlights	References
2014	5-FU	HT-29 cells (colorectal adenocarcinoma)	n.a.	β-CD-graft-gelatin crosslinked with oxidized dextran	Presence of β-CD increase drug loading. Swelling and drug release is low at pH 1.2 and high at pH 7.4, being appropriate for drug release at colon. Hydrogels were biocompatible and increase the efficacy of 5-FU.	[38]
2019	Bortezomib	MC3T3E1 (osteoblast) and NIH-3T3 (fibroblast) cells	n.a.	Alginate-conjugated polydopamine	The release mechanism followed non-Fickian diffusion. FACS analysis revealed cell apoptosis defined by loss of cell viability for colon cancer cells.	[39]
2019	Prospidin	HeLa (cervical adenocarcinoma) and HeP-2 (human hepatocellular liver carcinoma) cells	Zajdel hepatoma Mongrel white rats	Dextran phosphate (DP)	At low pH, the swelling of hydrogels is 4.6–12.3 times lower than at pH 7.4. Susceptible to degradation by the simulated physiological conditions. The amount of drug release is dependent on the pH of outer media and decreases with the growth of phosphoric group content in DP hydrogels	[40]
2015	5-FU	n.a.	n.a.	N-N′-dimethylacrylamide monomers polymerized in presence of methacrylic acid or 2-aminoethyl methacrylate hydrochloride containing ferro-nanoparticles	Drug release is always higher in the presence of a magnetic field and generally increases with its intensity.	[41]
2017	DOX	HepG2 cells (human hepatocellular liver carcinoma)	Sprague-Dawley rat	N-carboxyethyl chitosan + PEGDA	Exhibited in vitro pH-dependent gel degradation and doxorubicin release. No hydrogel diffusion after subcutaneous injection.	[33]
2019	PTX	HepG2 (human hepatocellular liver carcinoma), H22 (murine hepatoma)	H22 subcutaneous xenograft BALB/c mice	Self-assembling octapeptides	In vitro controlled release of PTX at pH 5.5 for 6 days. In vivo hydrogel retention at the tumor site. Increased antitumor efficacy compared to free PTX (reduced tumor weight and volume), and reduced toxicity (low weight loss).	[42]
2017	DOX	MDA-MB-231 cells (breast cancer)	n.a.	DNA hairpin conjugated with polyacrylamide	pH-induced separation of the nucleic acid duplex units causing DOX release at pH 5.0. Increase DOX cellular uptake and efficacy.	[43]
2019	DOX	HCT116 cells (colorectal adenocarcinoma)	Kunming Mice	Chitosan-grafted-dihydrocaffeic acid/oxidized pullulan	87% DOX release over 60 h at pH 5.5 over 52% at pH 7.4. In vitro enhancement of DOX therapeutic efficacy. In vivo adhesion in the injection site.	[44]
2019	5-FU and Rutin	MDA-MB-231 and MCF-7 cells (breast cancer)	n.a.	Zein, acrylic acid, N, N-methylene bisacrylamide, and ammonium persulphate	Improved release at pH 7.4 over pH 1.2. Improved in vitro pharmacological activity by apoptosis induction by oxidative stress.	[45]
2020	Triaryl-(Z)-olefin	n.a.	Ehrlich carcinoma cell subcutaneous xenograft Swiss albino mice	Cholesterol and span 60 niosomes in chitosan and glyceryl monooleate-based hydrogels	Controlled release of drug and improved tumor regression	[46]
2020	DOX	HeLa cells (cervical adenocarcinoma)	n.a.	Zein nanoparticle crosslinked pectin	Improved DOX cellular internalization and cytotoxicity	[47]
2020	PTX	A549 (lung cancer) and HepG2 (human hepatocellular liver carcinoma) cells	n.a.	Long-chain hexadecyl amine modified nanocellulose	Improved release at pH < 6.8. Improved PTX internalization by cells and therapeutic efficacy.	[48]
2020	DOX	HepG2 cells (human hepatocellular liver carcinoma)	HepG2 xenograft BALB/c nude mouse model	4armPEG-benzaldehyde and N-carboxyethyl chitosan	Degradation occurs at pH 5.6 which contributes for controlled DOX release. In vivo biocompatibility, degradation over 5 days, and improved tumor inhibition	[49]
2019	DOX	HeLa cells (cervical adenocarcinoma)	n.a.	Carboxyethyl modified chitosan and aldehyde modified hyaluronic acid	Improved drug release at pH < 6.8. Biocompatibility and biodegradability. In vitro pharmacological efficacy	[50]
